# The development and internal validation of a multivariable model predicting 6-month mortality for people with opioid use disorder presenting to community drug services in England: a protocol

**DOI:** 10.1186/s41512-024-00170-8

**Published:** 2024-04-16

**Authors:** Emmert Roberts, John Strang, Patrick Horgan, Brian Eastwood

**Affiliations:** 1 National Addiction Centre and the Department of Psychological Medicine, Institute of Psychiatry, Psychology and Neuroscience, King’s College London, London, UK; 2 South London and the Maudsley NHS Foundation Trust, London, UK; 3 Office for Health Improvement and Disparities, Department of Health and Social Care, London, UK

**Keywords:** Mortality, Opioid use disorder, Risk prediction, Prognostic modelling, Model development, Internal validation, England

## Abstract

**Background:**

People with opioid use disorder have substantially higher standardised mortality rates compared to the general population; however, lack of clear individual prognostic information presents challenges to prioritise or target interventions within drug treatment services. Previous prognostic models have been developed to estimate the risk of developing opioid use disorder and opioid-related overdose in people routinely prescribed opioids but, to our knowledge, none have been developed to estimate mortality risk in people accessing drug services with opioid use disorder. Initial presentation to drug services is a pragmatic time to evaluate mortality risk given the contemporaneous routine collection of prognostic indicators and as a decision point for appropriate service prioritisation and targeted intervention delivery. This study aims to develop and internally validate a model to estimate 6-month mortality risk for people with opioid use disorder from prognostic indicators recorded at initial assessment in drug services in England.

**Methods:**

An English national dataset containing records from individuals presenting to drug services between 1 April 2013 and 1 April 2023 (*n* > 800,000) (the National Drug Treatment Monitoring System (NDTMS)) linked to their lifetime hospitalisation and death records (Hospital Episode Statistics-Office of National Statistics (HES-ONS)). Twelve candidate prognostic indicator variables were identified based on literature review of demographic and clinical features associated with increased mortality for people in treatment for opioid use disorder. Variables will be extracted at initial presentation to drug services with mortality measured at 6 months. Two multivariable Cox regression models will be developed one for 6-month all-cause mortality and one for 6-month drug-related mortality using backward elimination with a fractional polynomial approach for continuous variables. Internal validation will be undertaken using bootstrapping methods. Discrimination of both models will be reported using Harrel’s *c* and *d*-statistics. Calibration curves and slopes will be presented comparing expected and observed event rates.

**Discussion:**

The models developed and internally validated in this study aim to improve clinical assessment of mortality risk for people with opioid use disorder presenting to drug services in England. External validation in different populations will be required to develop the model into a tool to assist future clinical decision-making.

## Introduction

In 2022, England reported its highest number of drug-related deaths on record (https://www.ons.gov.uk/peoplepopulationandcommunity/birthsdeathsandmarriages/deaths/bulletins/deathsrelatedtodrugpoisoninginenglandandwales/2021registrations). Almost half of all drug-related deaths involved an opioid whilst opioid use disorder was an issue for half of all adults accessing community drug services (https://www.gov.uk/government/statistics/substance-misuse-treatment-for-adults-statistics-2021-to-2022/adult-substance-misuse-treatment-statistics-2021-to-2022-report). Over the past decade, between 1 and 2% of all adults accessing community drug services with opioid use disorder died each year whilst receiving treatment (https://www.gov.uk/government/statistics/substance-misuse-treatment-for-adults-statistics-2022-to-2023/adult-substance-misuse-treatment-statistics-2022-to-2023-report). Professionals working in community drug services play a key role in delivering evidence-based care and support and in the provision of prognostic information to individuals with opioid use disorder. However, despite a good understanding that, on average, people with opioid use disorder have up to 10 times higher standardised mortality rates compared with the general population [1, 2], uncertainty regarding individual prognosis and mortality risk presents challenges to drug services in terms of providing individuals with accurate personalised risk information, prioritisation of finite resources and appropriate targeting of interventions.

Expansion in the use of clinical informatics and precision medicine has revolutionised the care provided in many healthcare sectors [3]; however, development and validation of prognostic risk models in populations of people with opioid use disorder has been relatively limited. This is despite multiple systematic reviews examining individual prognostic risk factors for mortality among people with opioid use disorder [1, 2, 4–6] and a number of studies recently developing models in populations routinely prescribed opioids (e.g. to examine the risk of developing opioid use disorder or the risk of opioid-overdose) [7, 8]. To our knowledge, no models have been explicitly developed examining mortality risk in people presenting to community drug services in those with a diagnosis of opioid use disorder. These could provide useful information and assistance to both individuals and professionals upon entering drug treatment to make collaborative treatment decisions.

Potential explanations for the relative paucity of prognostic modelling studies in this area include the required sample size and number of events and lack of centralised data repositories which include accurate prognostic and outcome information from healthcare and administrative agencies. England is unusual, having recently established a validated national data linkage between all hospitalisation, death and community drug treatment records [9]. This is coupled with the fact that all people in England, regardless of overseas visitor or immigration status, are able to access community drug services free of charge at the point of delivery and in the relative absence of a private treatment system [10]. The availability and coverage of this nationally linked dataset thus may provide a rare opportunity to develop and validate adequately powered prognostic models within this population.

### Objectives

This study will aim to develop and internally validate two models, one to estimate 6-month all-cause mortality risk and one to estimate 6-month drug-related mortality risk for people with opioid use disorder from prognostic indicators routinely recorded during initial assessment at community drug services in England.

## Methods

### Setting

The study utilises a national English dataset which contains linked individual records from two sources: (1) The National Drug Treatment Monitoring System (NDTMS)—a centralised database, collated and maintained by the Department of Health and Social Care (DHSC), which receives monthly input from all adult statutory community drug services in England [11]. NDTMS contains individual-level data on an individual’s sociodemographic characteristics (date of birth, sex, ethnicity, housing status, etc.), what substances the individual is using problematically, any treatment interventions received and measures of treatment success. (2) Hospital Episode Statistics-Office of National Statistics (HES-ONS)—a centralised database, collated and maintained by the National Health Service (NHS), which collects all information pertaining to NHS inpatient hospitalisation in England [12]. HES-ONS covers all NHS inpatient admissions, including any admission to private or third-sector hospitals subsequently reimbursed by the NHS, and is estimated to contain > 99% of all inpatient hospital activity in England. An inpatient hospital admission includes any secondary care-based activity requiring a hospital bed, thus includes day cases, and both planned and emergency admissions, in physical and mental health settings. HES-ONS does not cover accident and emergency (A&E, emergency department) attendances, nor outpatient bookings, these data being held in separate databases. In addition, HES-ONS contains official death certification records for those individuals who have died. The overall structure of the linked NDTMS-HES-ONS data is clustered with individuals attending one of 150 uniquely commissioned drug and alcohol services across each local authority area in England.

Approval to conduct the linkage analysis was granted under regulation 3 of the Health Service (Control of Patient Information) Regulations 2002, following review by the Caldicott Advisory Panel (CAP) (Ref: CAP-2019–06) and the Department of Health and Social Care Office of Data Protection (ODP). NDTMS data are available from 1 April 2013 to 1 April 2023, containing data on *n* > 800,000 unique individuals over the age of 18 who presented to community drug treatment at least once within that timeframe. Linked HES-ONS data is available for these all individuals detailing any subsequent death records and any individual hospital admissions since the HES database inception in 1997 [9]. The database can only be accessed by DHSC staff working on the project with all records stored for a minimum of 5 years after study completion. This study protocol has been designed in accordance with the TRIPOD statement for transparent reporting of the development of multivariable predictive models [13] and has been co-developed with input from the South London and the Maudsley Biomedical Research Centre Data Linkage Service User and Carer Advisory Group which includes experts with lived experience of opioid use disorder [14].

### Candidate indicator variables

The prognostic indicators for consideration in the multivariable model were identified from multiple systematic reviews and underlying included studies which examined demographic and clinical features associated with increased mortality for people with opioid use disorder [1, 2, 4–6, 15]. All prognostic indicator variables are extracted from NDTMS-HES-ONS records retrospectively from the time of initial assessment at the community drug service using the date of the most recent initial assessment as time zero (*t*0). Given the aim is to create a model that could be readily incorporated into routine clinical care within time-pressured drug services, a parsimonious approach was taken to a selection of prognostic indicators with clinician and patient involvement suggesting that, ideally, no more than ten variables should be included in a final model. Twelve candidate prognostic indicator variables were initially identified; their descriptions and variable structure can be found in Table 1 (https://digital.nhs.uk/data-and-information/data-tools-and-services/data-services/hospital-episode-statistics/hospital-episode-statistics-data-dictionary), (https://www.gov.uk/government/publications/national-drug-treatment-monitoring-system-reference-data).

**Table 1:** Candidate predictor variables

Candidate Predictor Variable	Variable structure in NDTMS-HES-ONS
Age	Continuous
Sex	Binary: 1: Male 2: Female
Injecting status	Categorical: 1: Currently injecting 2. Previously injected (but not currently) 3: Never injected
HIV positivity	Binary: 1: Yes 2: No
Hepatitis C RNA positivity	Binary: 1: Positive 2: Negative (never infected or cleared by treatment)
Polysubstance use: Current number of substances used problematically	Count
Problematic alcohol use	Binary: 1: Any current problematic use of alcohol 2: No current problematic use of alcohol
Problematic benzodiazepine use	Binary: 1. Any current problematic use of any benzodiazepine 2. No current problematic use of any benzodiazepine
Housing status	Categorical: 1: No fixed abode—urgent housing problem, i.e -—Lives on streets/rough sleeper -—Uses night shelter (night-by-night basis) /emergency hostels -—Sofa surfing/sleeps on different friend’s floor each night 2: Housing problem, i.e - Staying with friends/family as a short-term guest - Night winter shelter - Direct access short stay hostel - Short-term B & B or other hotel - Placed in temporary accommodation by the Local Authority - Squatting 3: No housing problem - Owner-occupier - Tenant—private landlord/ housing association/ Local - Authority/registered landlord/arm’s length management organisation - Approved premises - Supported housing/hostel - Traveller - Own property - Settled mainstream housing with friends/family - Shared ownership scheme
Recent acute inpatient hospital admission	Binary: 1. Inpatient acute hospital admission within the last 6 months 2. No acute inpatient hospital admissions within the last 6 months
Recent mental health inpatient hospital admission	Binary: 1. Inpatient mental health hospital admission within the last 6 months 2. No inpatient mental health hospital admissions within the last 6 months
Recent history of incarceration	Binary: 1. Referred to the drug service from prison 2. Referred to the drug service by any other source

### Outcome measures

The binary outcomes of all-cause and drug-related mortality will be assessed prospectively for each individual at 6 months after* t*0, this timepoint chosen following clinician, patient and public involvement feedback. Drug-related death follows the definition used by the ONS when reporting official national statistics for deaths related to drug poisoning. The included death certificate International Classification of Diseases, Tenth Revision (ICD-10) codes for drug-related death can be found in Table 2.

**Table 2:** International Classification of Diseases, Tenth Revision (ICD-10) codes used to define drug-related deaths

Description	ICD-10 Codes
Mental and behavioural disorders due to drug use (excluding alcohol and tobacco)	F11–F16, F18–F19
Accidental poisoning by drugs, medicaments and biological substances	X40–X44
Intentional self-poisoning by drugs, medicaments and biological substances	X60–X64
Assault by drugs, medicaments and biological substances	X85
Poisoning by drugs, medicaments and biological substances, undetermined intent	Y10–Y14

### Sample size

The minimum required sample size for time-to-event model development is based on estimated event rates of the prediction model outcomes [16]. Given that the drug-related death event rate is by definition smaller than the all-cause death rate, and thus requires a larger sample size, this outcome was chosen for sample size calculation. Estimation used the ‘pmsampsize’ command, and in the absence of any reported Cox-Snell *R*-squared values from previously developed models, we aimed to develop a model with a minimal anticipated Harrel’s *c*-statistic (a measure of discrimination similar to the area under a receiver operating characteristic (ROC) curve but taking account of the censored nature of the data) of 0.70, allowing a maximum shrinkage of 10% to minimise potential overfitting [17]. A maximum total of 12 candidate predictors is planned with an estimated event rate based on a previous cohort study which reported 0.0134 drug-related deaths per person-year [15]. This estimated a minimum required sample size of 2487 participants and 51 events.

### Missing data

The proportion of missing data and its assumed missingness mechanism will be assessed and reported for each candidate predictor variable. Where appropriate, and if the missing at random (MAR) assumption is met, missing data will be addressed using multiple imputation by chained equations (MICE) [18]. The number of imputations is determined using the fraction of missing information (FMI) for each predictor such that the number of imputations is equal to the proportion of the FMI, i.e. 20 imputations if the FMI is 0.2 [18].

### Statistical analysis

Multivariable Cox regression will be used for model development with complete outcome data available for all participants at 6 months [19]. The model will be developed through backward elimination with the level of alpha for variable exclusion set at 0.157, as recommended based on the Akaike Information Criterion (AIC) [20]. Nonlinearity of continuous variables will be addressed by using a multivariable fractional polynomial approach, an established technique for transforming non-linear continuous variables when developing a backward elimination model [21]. Model discrimination will be assessed through the calculation of Harrel’s *c* and *d*-statistics and calibration curves and slopes will be presented and the ratio of the observed to predicted event rates examined [21, 22]. Internal validation will be undertaken using bootstrapping resampling methods, which account for bias due to over-fitting more accurately than split-sample cross-validation approaches, with the model development process repeated in 1000 bootstrap samples to allow calculation of optimism adjusted discrimination and calibration measures [23]. Performance will also be evaluated by calculation of Harrell’s *C* statistics for each cluster (i.e. each of the 150 individual drug and alcohol services) and the results combined using random effects meta-analysis. Between-cluster heterogeneity will be assessed using the *I*^2^ statistic with a derivation of 95% prediction intervals for performance measures [22]. Other potentially complimentary analytic techniques, including decision curve analysis, will also be explored. All analyses will be conducted in Stata version 18.0 (StataCorp, College Station, TX, USA), with full reporting of how the final prediction model was developed. We will report the final multivariable model equation including estimation of the baseline hazard function.

## Discussion

This protocol aims to describe the rationale and methods to develop and internally validate a prognostic risk model to estimate 6-month all-cause and drug-related mortality for people with opioid use disorder presenting for an initial assessment at community drug services in England. To our knowledge, no previous models have been developed examining these outcomes in the studied population, which may provide clinically useful information and assistance to both patients and professionals when making treatment and care decisions in community drug services.

There are multiple strengths to the proposed study including the comprehensive and national nature of the dataset and the involvement of clinicians and patients from the outset to consider variable, outcome and overall model utility. Whilst pre-publication of the study protocol and commitment to adherence to transparent reporting guidelines additionally strengthen the study, there are several potential limitations [13]. All prognostic indicator variables will be collected retrospectively from an administrative dataset the underlying data for which has been supplied by drug treatment services. There is therefore a risk of lack of availability of some variables if submitted documentation is incomplete, with a detailed assessment of potential missingness mechanism crucial. Whilst relying on routinely documented clinical information as the source of prognostic information has limitations, this approach has been utilised frequently and does reflect how the model would likely be used in clinical practice, with some information potentially not being available to professionals or patients at the time of initial assessment. The model will require independent external validation in other samples, with potentially suitable datasets identified in both Wales and Australia [24, 25], and subsequent examination of its utility in clinical practice and acceptability among professional and patient groups. Continued co-production through development, validation and implementation with both clinicians and patients will remain a key requirement.

Whilst there have been significant expansions and understanding in the use of machine learning methods to develop prognostic models across healthcare sectors, initial patient and public involvement work with service users and clinicians demonstrated reticence to employ these within the context of mortality prediction in opioid use disorder. The perception of a ‘black box’ or lack of transparent understanding of what prediction outcome scores were based on, and the relative infancy of clinical informatics within the opioid use disorder space led to concerns about clinical utility, and implementation within community drug services. Clinicians working within drug services were comfortable with clinical risk tools developed using classical statistical methods, and their corollaries used in other areas of healthcare [26], and welcomed their potential expansion within addiction settings. However, there was concern among service users that results from machine learning methods would not be believed, and explanation of algorithms could create difficulties in conveying the predictive information to individuals accessing drug services. As such, traditional statistical methods were chosen to develop this initial protocol.

Standardised all-cause and drug-related mortality rates are significantly elevated among people with opioid use disorder, and despite a significant body of literature describing individual prognostic risk factors, often clinical judgement alone is used to consider prognosis and the prioritisation of treatment interventions in drug treatment services. Whilst other areas of medicine routinely incorporate risk tools into care to assist clinical decision making [26], clinical informatics within the addiction field has been somewhat slower to progress. Given the significant elevated mortality risks within this population, the development of accurate prognostic models appears timely, warranted and urgent. Notwithstanding these observations, it is vital any developed model is validated, demonstrates clinical utility and has buy-in from both professionals and patients if it is to be valued and successfully implemented.

## Data Availability

The datasets generated and analysed during the current study are not publicly available as they contain sensitive patient identifiable data. Whilst access to the linked dataset is only available within DHSC, subject to approval, extracts of NDTMS are available to researchers, and extracts of HES-ONS mortality are available through the Data Access Request Service (DARS) at NHS England.

## Abbreviations

AIC: Akaike Information Criterion
CAP: Caldicott Advisory Panel
DHSC: Department of Health and Social Care
FMI: Fraction of Missing Information
HES-ONS: Hospital Episode Statistics-Office of National Statistics
HIV: Human immunodeficiency virus
ICD: International Classification of Disease
MAR: Missing at random
MICE: Multiple imputation by chained equations
NDTMS: National Drug Treatment Monitoring System
NHS: National Health Service
ODP: Office of Data Protection
ROC: Receiver operating characteristic
